# Supplementary material for: The abrogation of condensin function provides independent evidence for defining the self-renewing population of pluripotent stem cells
Source: Dev Biol. 2018 Jan 15;433(2):218–26. doi: 10.1016/j.ydbio.2017.07.023 (PMC5771471; doi:10.1016/j.ydbio.2017.07.023)
Supplement: Supplementary file 2 — Supplementary material List of primers used in PCR and cloning. [file mmc5.pdf]

Table S1

Gene	Forward primer	5'-3' sequence	Reverse primer	5'-3' sequence	length (bp)
NCAPG	1F	CATTACCATCCCGCGGCAATGGTAATGAGGATA	1R	CCAATTCTACCCGTCGCCGTTAAATACTACCAG	391
NCAPG	2F	CATTACCATCCCGGTCATCGAAACTGTTGTCAC	2R	CCAATTCTACCCGCCGATATTAGCAAGCTTCCT	865
NCAPD2	3F	CATTACCATCCCGTGTTGCCTCACATTTGCTCTAAAG	3R	CCAATTCTACCCGATTGATTGCAGTCCCAATGGTTTT	720
NCAPH	4F	CATTACCATCCCGAATGAAAAGATGCTCGGATTGTGG	4R	CCAATTCTACCCGAATTGACGCTTGTAAAATGGACC[illegible]	717
SMC2	5F	CATTACCATCCCGAACAGAAACAATTGGCCACTGAAA	5R	CCAATTCTACCCGAAACGATCGGCATTTTCTTTCTGT	757
SMC4	6F	CATTACCATCCCGCGGAGATACATTGGCTGAGTATCA	6R	CCAATTCTACCCGCAAGAGCCAATGATGAAAGGGTT	785

Sequences marked in red are added for pT4P directional cloning
